# Nutritional Assessment in Adult Patients with Dysphagia: A Scoping Review

**DOI:** 10.3390/nu13030778

**Published:** 2021-02-27

**Authors:** Junko Ueshima, Ryo Momosaki, Akio Shimizu, Keiko Motokawa, Mika Sonoi, Yuka Shirai, Chiharu Uno, Yoji Kokura, Midori Shimizu, Ai Nishiyama, Daisuke Moriyama, Kaori Yamamoto, Kotomi Sakai

**Affiliations:** 1 Department of Clinical Nutrition and Food Services, NTT Medical Center Tokyo, 5-9-22 Higashi-Gotanda, Shinagawa, Tokyo 141-8625, Japan; 2 Department of Rehabilitation Medicine, Mie University Graduate School of Medicine, 2-174 Edobashi, Tsu Mie 514-8507, Japan; momosakiryo@gmail.com; 3 Department of Nutrition, Hamamatsu City Rehabilitation Hospital, 1-6-1 Wago-kita, Naka-ku, Hamamatsu, Shizuoka 433-8127, Japan; a.shimizu.diet@gmail.com; 4 Tokyo Metropolitan Institute of Gerontology, 35-2, Sakaecho, Itabashi-ku, Tokyo 173-0015, Japan; kemotokawa@gmail.com (K.M.); yamamoto0915093@gmail.com (K.Y.); 5 Department of Clinical Nutrition, Okayama University Hospital, 2-5-1, Shikatacho, Kita-ku, Okayama 700-8558, Japan; mika.sonoi@gmail.com; 6 Department of Nutrition, Hamamatsu University School of Medicine, 1-20-1 Handayama, Higashi-ku, Hamamatsu, Shizuoka 431-3192, Japan; yunnkaa@gmail.com (Y.S.); wadamido0917@gmail.com (M.S.); 7 Institutes of Innovation for Future Society, Nagoya University, 65 Tsurumaicho, Showa-ku, Nagoya, Aichi 466-8560, Japan; chiharu83724@gmail.com; 8 Department of Clinical Nutrition, Keiju Medical Center, 94 Tomiokacho, Nanao, Ishikawa 926-8605, Japan; yojikokura@hotmail.com; 9 Department of Clinical Nutrition and Food Service, Yasuoka Hospital, 3-16-35, Yokono-cho, Shimonoseki, Yamaguchi 759-6604, Japan; ai.htks.n@gmail.com; 10 Department of Nutrition, Chubu Rosai Hospital, 1-10-6 Koumei, Minato-ku, Nagoya, Aichi 455-8530, Japan; moriyama.nut@gmail.com; 11 Department of Rehabilitation Medicine, Setagaya Memorial Hospital, 2-30-10 Noge, Setagaya, Tokyo 158-0092, Japan; koto.sakai1227@gmail.com

**Keywords:** swallowing, malnutrition, nutritional status, GLIM, adults

## Abstract

Malnutrition negatively affects the quality of life of patients with dysphagia. Despite the need for nutritional status assessment in patients with dysphagia, standard, effective nutritional assessments are not yet available, and the identification of optimal nutritional assessment items for patients with dysphagia is inadequate. We conducted a scoping review of the use of nutritional assessment items in adult patients with oropharyngeal and esophageal dysphagia. The MEDLINE, EMBASE, and Cochrane Central Register of Controlled Trials databases were searched to identify articles published in English within the last 30 years. Twenty-two studies met the inclusion criteria. Seven nutritional assessment categories were identified: body mass index (BMI), nutritional screening tool, anthropometric measurements, body composition, dietary assessment, blood biomarkers, and other. BMI and albumin were more commonly assessed in adults. The Global Leadership Initiative on Malnutrition (GLIM), defining new diagnostic criteria for malnutrition, includes the categories of BMI, nutritional screening tool, anthropometric measurements, body composition, and dietary assessment as its required components, but not the blood biomarkers and the “other” categories. We recommend assessing nutritional status, including GLIM criteria, in adult patients with dysphagia. This would standardize nutritional assessments in patients with dysphagia and allow future global comparisons of the prevalence and outcomes of malnutrition, as well as of appropriate interventions.

## 1. Introduction

Dysphagia is a global health problem estimated to affect 8% of the world’s population [1]. Dysphagia diminishes the quality of life of individuals [2,3], and dysphagia patients who are malnourished and who do not have access to appropriate treatment and interventions sustain a longer hospital stay, higher risk of complications, and higher mortality rate than those who are properly nourished [4,5]. Therefore, dysphagia and malnutrition are closely associated [6,7]. It is reported that 39.2% of dysphagic patients are at risk for malnutrition and that 13.6% of individuals at risk for malnutrition have dysphagia [8]. Besides, the prevalence of concurrent malnutrition and dysphagia has been estimated between 3% and 29% [9,10]. Patients with oropharyngeal dysphagia (OD) are prone to receiving inadequate food intake and presenting malnutrition because of fear of choking, anorexia, and decreased food preference related to food texture [11]. In addition, texture-modified diets are lower in nutrients than a regular diet and are more likely to induce malnutrition and sarcopenia than a regular diet [12,13,14]. Malnutrition leads to systemic muscle mass loss and atrophy of the muscles used to swallow, and this ultimately leads to dysphagia [15,16]. Therefore, it is recommended that the nutritional status of all dysphagic patients should be assessed [17,18].

Nutritional assessment is the process of determining if there is a problem with an individual’s nutritional status, identifying it, and performing a detailed examination to determine the severity of malnutrition [19]. A nutritional assessment must also include variables that will help in the appropriate follow-up of the patient after nutritional therapy has been implemented [20]. Specifically, it includes the evaluation of subjective and objective parameters, such as medical history, dietary intake, physical examination, anthropometric measurements, physical function, mental function, quality of life, medications, and laboratory data [21,22]. Namasivayam et al. [9] conducted a systematic review of the impact of dysphagia on malnutrition in patients in long-term care. Body mass index (BMI), weight loss, Mini Nutritional Assessment (MNA), and laboratory data (serum and urinary tests) were identified as indicators in a nutritional assessment, but there was no uniformity in their review. In addition, for BMI, which was the most commonly used measure in the studies reviewed, different cutoff values were chosen. Namasivayam et al. concluded that it was difficult to accurately ascertain the prevalence of malnutrition due to discrepancies in the measurement methods used. The Global Leadership Initiative on Malnutrition (GLIM) was advocated by several of the global clinical nutrition societies in 2018, with the aim of enabling global comparisons of the prevalence of malnutrition and related interventions and outcomes [23,24]. However, the optimal nutritional assessment items for dysphagia patients have not yet been identified. Deeper knowledge in this area will facilitate the identification of the optimal nutritional assessment items for patients with dysphagia and help us to understand the actual prevalence of malnutrition in these patients. This may allow us to spread awareness on the issue of malnutrition and facilitate early nutritional interventions in dysphagia patients. As a result, it may be possible to prevent a reduction in the quality of life caused by malnutrition in dysphagia patients. The aim of this scoping review was to identify the most important items to include in the nutritional assessment for patients with dysphagia.

## 2. Materials and Methods 

We conducted a scoping review to answer the following research question: “What are the appropriate nutritional assessment items for adult patients with oropharyngeal and esophageal dysphagia?” This scoping review protocol was registered in advance [25]. Scoping reviews are conducted to map out key concepts underlying a research area, the main sources of information, types of study, and evidence available and to clarify the definitions and conceptual boundaries of a topic [26]. In other words, they aim at (i) identifying types of available evidence in a given field, (ii) identifying and analyzing knowledge gaps, (iii) clarifying key concepts/definitions in the literature, (iv) examining how research is conducted in a certain topic or field, and (v) identifying key characteristics or factors associated with a concept [27]. The most common reasons for conducting scope reviews are to explore the breadth and scope of the literature, map and summarize the evidence, and inform future research [28]. Scope reviews can be conducted as a preliminary exercise before conducting systematic reviews [29], and unlike systematic reviews, the process of assessing the risk of bias and synthesizing findings from individual studies to generate “summary” findings is not mandatory [27]. We used a scoping review methodology consistent with the Joanna Briggs Institute’s guidance [30] to ensure clarity and rigor in the review process. Additionally, our review was performed in accordance with the scoping review reporting guidelines of the PRISMA Extension for Scoping Reviews (PRISMA-ScR) [31]. 

### 2.1. Eligibility Criteria

This scope review included studies in which nutritional assessment was performed on adult patients with dysphagia. The concepts examined in this scoping review included components of nutritional assessment in the adult population with dysphagia. Components of the nutritional assessment were “Nutritional Assessment,” “Nutritional Status,” “Body Composition,” and “Dietary Assessment” [21,32,33]. Individuals with dysphagia were defined as those clinically diagnosed with dysphagia by videofluoroscopy swallowing studies or fiberoptic endoscopic swallowing assessment [34]. The inclusion criteria were observational and intervention studies in which nutritional assessment was performed in adult patients with oropharyngeal and esophageal dysphagia. In addition, articles and literature in textbooks and peer-reviewed journals written in English and published between January 1991 and May 2020 were eligible for inclusion. Review articles, studies not including 100% of patients with dysphagia, studies using animal models, qualitative studies, case reports, and conference abstracts were excluded.

### 2.2. Search Strategy

**Figure 1:**
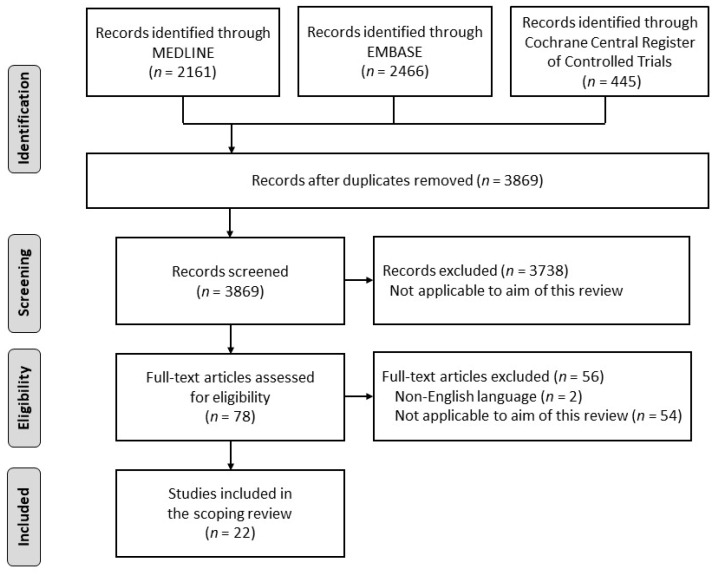
Flowchart of the search strategy. An initial search of studies was performed using the MEDLINE, EMBASE, and Cochrane Central Register of Controlled Trials databases (*n* = 5072). Screening for eligibility and full-text assessments were performed. Twenty-two articles were included in the final analysis.

The first step in the search strategy consisted in conducting a pilot search in MEDLINE to identify articles on this topic. Text words in the titles and abstracts and the index terms used to describe these articles were used to develop the search strategy. Next, the search formula developed was used to search three databases (MEDLINE, EMBASE, and Cochrane Central Register of Controlled Trials). The most recent search was performed on 12 May 2020. The search strategy was presented in the protocol registration in advance [25]. The references searched were imported into Rayyan version 0.1.0 (Qatar Computing Research Institute, Doha, Qatar; https://rayyan.qcri.org/ (accessed on 12 May 2020)). Twelve reviewers used Rayyan to screen the titles and abstracts of articles for eligibility and to assess their full text. Disagreements between reviewers were resolved through discussion, and an independent reviewer was consulted when necessary to resolve any disagreements. An overview of the scoping review process is shown in Figure 1.

### 2.3. Data Extraction

A single reviewer extracted data from the articles that satisfied the eligibility criteria. Narrative synthesis was used in the data analysis to characterize the studies analyzing nutritional assessments in patients with dysphagia. The extracted data were analyzed according to the following variables: setting, characteristics of the patients with dysphagia (age and comorbidities), nutritional assessor, nutritional assessment items, and author(s), year, and country of the publication (Table S1).

## 3. Results

In the initial search, we identified 5072 articles using the MEDLINE, EMBASE, and Cochrane Central Register of Controlled Trials databases. Duplicates were removed, the titles and abstracts of these articles were screened for eligibility, and their full texts were assessed. After applying the exclusion criteria (Table S2), twenty-two articles were included in the analysis (Figure 1). The nutritional assessment items from the included articles were categorized into seven categories: BMI, nutritional screening tool, anthropometric measurements, body composition, dietary assessment, blood biomarkers, and other. The assessors included professionals, dietitians, multidisciplinary teams (Table S1).

### 3.1. Nutritional Assessment in Adult Patients with Dysphagia 

Table 1 shows the nutritional assessment items for adult patients with oropharyngeal and esophageal dysphagia identified in this review. The most frequently measured nutritional assessment in adults was BMI, which was recognized in nine articles. It was assessed with major diseases that cause dysphagia. The MNA short form (MNA-SF) was recognized as a nutritional screening tool in four articles. The most common anthropometric measurement was body weight, recognized in five articles, followed by triceps skinfold thickness (TSFT) and midarm muscle circumference (MAMC). Body composition was measured using the bioelectric impedance analysis (BIA) method; percent body fat was measured in one article. No articles measured body composition using dual-energy X-ray absorptiometry. For dietary assessment, two articles assessed the food intake level through the Food Frequency Questionnaire, evaluating energy, daily food intake, and dietary form. MNA and Onodera’s Prognostic Nutritional Index were included in the “other” category. Several articles used only one indicator to assess the nutritional status. Two articles used only BMI (da Silva et al. [35] and Ikenaga et al. [36]), two articles used only weight (Kim et al. [37] and Wang et al. [38]), one article assessed nutrition using only the nutrition screening tool (Vilardell et al. [39]), and one article (Masiero et al. [40]) reported data on daily food intake. The majority of the participants in the studies included in the present scope review were older adults (Table S1).

### 3.2. Blood Biomarkers from Nutritional Assessment in Adult Patients with Dysphagia 

Blood biomarkers included in the nutritional assessment of adults are shown in Table 2. The most common item was albumin (nine articles). Serum visceral proteins, such as pre-albumin, transferrin, and retinol-binding protein, were also measured. Albumin was measured mostly in patients with stroke dysphagia. Two articles assessed nutrition based on blood biomarkers alone: Miyake et al. [55] assessed total protein and albumin levels, and Kimura et al. [56] assessed albumin and lymphocytes levels.

### 3.3. Comparison of Nutritional Assessment Items for Patients with Dysphagia in Acute and Postacute Settings

Nutritional assessment items were categorized by setting (Table 3). We classified and reviewed the clinical situations as acute settings (e.g., acute care hospitals) and post-acute settings (nonacute settings such as rehabilitation hospitals and clinics) because a previous report [57] exists on the nutritional status of OD patients in different clinical situations (chronic vs. acute). The acute setting included six of the seven categories identified in this study, except for “other”. Moreover, the dietary assessment, in particular, was an item that allowed a detailed assessment of the daily nutritional intake and form. Blood biomarkers with short half-lives (e.g., transthyretin, pre-albumin, and transferrin) were often used. In contrast, the post-acute setting included categories other than anthropometric measurements among the seven categories. Consequently, the number of items used in each nutritional assessment category was fewer. BMI, MNA-SF, and albumin were used in both settings. The diseases in each setting showed a mixture of acute and chronic dysphagia. 

## 4. Discussion

Three conclusions were achieved with this scoping review. First, the nutritional assessment items for patients with dysphagia were categorized into seven categories, and BMI was one of the most commonly used nutritional assessment item. Second, serum visceral proteins were commonly used as blood biomarkers items, with albumin being the most frequently used. Third, BMI, MNA-SF, and albumin were items that could be used regardless of the setting. Consequently, this study was able to identify several additional nutritional assessment items that were characteristic of the study setting.

**Figure 2:**
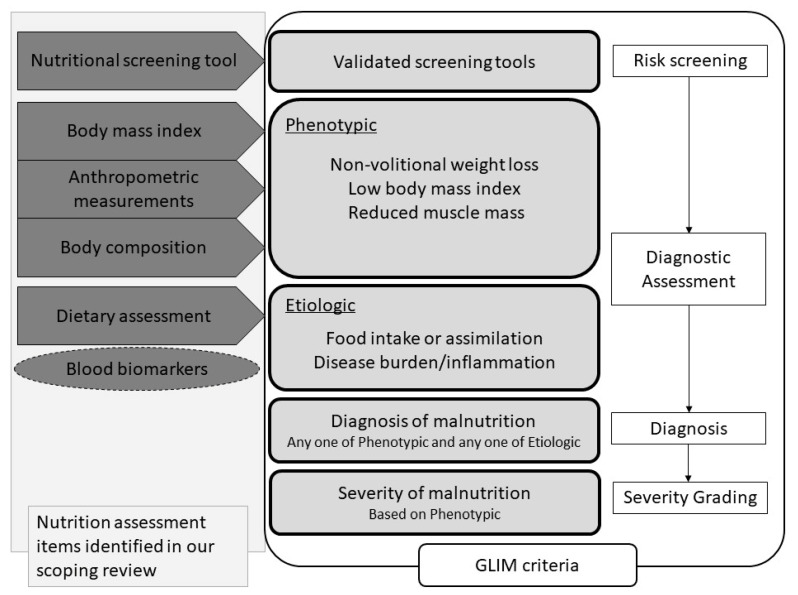
Flowchart for nutritional diagnosis using the GLIM criteria and nutritional assessment components. The nutritional assessment items identified in this review are listed on the far left. Among the items identified in this study, the components of the GLIM criteria are nutritional screening tools, BMI, anthropometric measurements, body composition, and dietary assessment. Blood biomarkers are surrounded by a dotted line because the GLIM criteria recommend their use as adjunct indicators of disease burden/inflammation. Abbreviations: GLIM, Global Leadership Initiative on Malnutrition.

BMI was one of the most commonly used items in nutritional assessment. BMI is generally used as a common indicator of malnutrition [58]. Although many global regions use BMI as a criterion for determining malnutrition [59,60,61,62], overweightedness and obesity are more of a problem in North America, including in the United States, than a low BMI [23]. Therefore, BMI is not used necessarily as a marker of clinical malnutrition [23]. In addition, the percentages of lean fat mass and fat mass in the body are not determined by the BMI. Sarcopenia is found in obese and nonobese individuals and is an important health problem for the older population, leading to poor prognosis in terms of physical dysfunction, poor quality of life, and increased mortality [63]. Therefore, in older adults, not only BMI but also muscle mass and muscle function should be assessed [64]. Sarcopenia also causes dysphagia [16,65]; therefore, muscle mass, muscle strength, and physical function should also be assessed in addition to BMI. The GLIM criteria [23,24], which are new malnutrition diagnostic criteria, may be suitable for assessing nutrition in adults with dysphagia, because they can assess both muscle mass and BMI. The components of the GLIM criteria include the nutritional screening tool, BMI, anthropometric measurements, body composition, dietary assessment, and impact of disease, and these criteria contain five of the seven categories identified in this review (Figure 2). Various diagnostic criteria for malnutrition exist (e.g., Subjective Global Assessment [66], American Society for Parenteral and Enteral Nutrition/Academy of Nutrition and Dietetics 2012 [67], and European Society for Clinical Nutrition and Metabolism 2015 [59]). However, none of them include all items of the nutritional screening tool, BMI, anthropometric measurements, body composition, dietary assessment, and impact of the disease. MNA was recognized in this study to consider nutritional screening tools, BMI, anthropometric measurements, body composition, dietary assessment, and impact of the disease, but its indications are for older adults. The use of MNA may be limited for patients with dysphagia in a wide age group.

Serum visceral proteins were commonly used as blood biomarkers items. Of these, albumin was the most frequently used. Albumin is one of the biochemical indices that decrease during malnutrition. Still, in periods of acute illness, the hepatic production of proteins such as albumin, prealbumin, and transferrin is downregulated, resulting in lower levels in the serum [68,69]. Therefore, these proteins can have a low serum concentration independent of the actual nutritional status [11] and should be interpreted with caution in patients with infections, acute inflammation, and trauma [70]. Dysphagic patients are at high risk for developing pneumonia, which is often an acute inflammatory condition. Evans et al. [71] propose that visceral proteins should not be used as nutrition markers because they characterize inflammation rather than describe the nutrition status. In the GLIM criteria, the albumin level is also a useful reference for a patient’s inflammatory status, but it is not included as a component of the diagnosis (Figure 2). For these reasons, nutritional assessments using only albumin, prealbumin, or transthyretin are not appropriate for dysphagia patients who are prone to acute inflammation such as pneumonia. We suggest that blood biomarkers should not be used as nutritional assessments by themselves, but they should rather be employed as an adjunct or additional indicator to the nutritional assessment.

In the list of nutritional assessment items by setting (Table 3), BMI, MNA-SF, and albumin were used in acute and post-acute settings. Therefore, on the one hand, BMI and MNA-SF can be used as nutritional assessment items for patients with dysphagia. On the other hand, albumin can be used as an adjunct indicator for nutritional assessment, regardless of the setting. Moreover, several unique nutritional assessment items have been identified depending on the study setting. In the acute setting, items that can be used to assess nutritional intake and form in detail were used as dietary assessment items, in contrast to the post-acute setting, suggesting that daily food intake, period to meal resumption, and dietary form may be used as short-term nutritional indicators. In addition, the impact of the inflammatory response may need to be more strongly considered in the acute setting. In a previous study [57] that examined the differences in the nutritional status of OD patients in acute and chronic situations, OD patients in chronic situations presented with malnutrition, sarcopenia, reduced visceral and muscular protein compartments and fat compartments, muscle weakness, intracellular water depletion, and weight loss. Patients in acute situations also presented with malnutrition and sarcopenia, but also showed more severe reductions in serum visceral protein and muscle mass due to the inflammatory response to pneumonia. The current scoping review also assessed visceral proteins with a short half-life and C-reactive protein (CRP) in the acute setting. This suggests that the evaluation of visceral protein and CRP is essential in addition to the evaluation of malnutrition in patients with OD in the acute setting. Furthermore, assessing sarcopenia and dehydration in addition to malnutrition may be necessary in the chronic setting, as reported by Carrión et al. [57], although sarcopenia and dehydration were not assessed in the post-acute setting in this scoping review. 

Although none of the articles in this review used the GLIM criteria to diagnose malnutrition, we recommend using the GLIM criteria initially for adult patients with dysphagia. The reasons are that the GLIM criteria can assess both muscle mass and BMI and can determine the effects of a disease [23,24]. It is essential to consider the impact of acute or chronic diseases in the nutritional assessment of dysphagia patients, such as post-acute stroke and neuromuscular diseases. One of the advantages of the GLIM criteria is that they consider the impact of disease, such as whether an inflammatory condition is acute disease-, injury-, or chronic disease-related. An association between GLIM-defined malnutrition and post-stroke dysphagia has already been reported [72]; however, the association between GLIM-defined malnutrition and dysphagia caused by other diseases has not been analyzed. As mentioned above, malnutrition in patients with dysphagia is influenced not only by the disease itself but also by background diseases. Therefore, the prevalence of malnutrition may vary for each disease that causes dysphagia. However, due to differences in the nutritional assessment methods used, the current actual prevalence of malnutrition is difficult to determine [9,11]. This makes it difficult to develop and compare effective intervention methods. The GLIM criteria were developed to disseminate the use of standardized assessment items for comparing the prevalence of malnutrition and intervention methods globally. However, the GLIM criteria can be used for risk screening and malnutrition diagnosis, but not for a detailed comprehensive nutritional assessment [23,24]. Therefore, the nutritional assessment of adults should be carried out using the GLIM criteria at a minimum, and additional comprehensive nutritional assessments should be conducted in the presence of malnutrition. Based on the results of this review, patients with OD need to be assessed for visceral protein and CRP in addition to the assessment of malnutrition in the acute setting. However, sarcopenia and dehydration may need to be assessed in addition to malnutrition in the chronic setting. This will enable us to identify the real prevalence of malnutrition in adult patients with dysphagia and to develop and compare effective interventions.

There are several limitations to this review. First, because it includes only articles with 100% of the participants being dysphagia patients, there is no recognition of the nutritional assessments used in studies comparing these patients with individuals without dysphagia. The results of the review may change when this is accounted for. Second, only three databases were used for the literature search. Although we implemented a rigorous search and review process, some relevant manuscripts may not have been considered because of the database selection, search strategy, and article selection method. Third, this study was not able to strictly distinguish between acute and chronic situations in patients with dysphagia, as Carrión et al. did [57]. However, the study setting (acute and post-acute) was used as a reference to classify the patients. Fourth, this study focused on patients with oropharyngeal dysphagia and esophageal dysphagia. However, the current study was not able to sufficiently examine the nutritional assessment items in relation to these two different types of dysphagia because only two papers on esophageal dysphagia were found. (References [73,74,75,76,77,78,79,80,81,82,83,84,85,86,87,88,89,90,91,92,93,94,95,96,97,98,99,100,101,102,103,104,105,106,107,108,109,110,111,112,113,114,115,116,117,118,119,120,121,122,123,124,125,126] are cited in the “Supplementary Materials”).

## 5. Conclusions

We identified nutritional assessment items for adult patients with oropharyngeal and esophageal dysphagia in this scope review. We found that various nutritional assessment items were used, making it difficult to confirm the real nutritional status of patients with dysphagia. Therefore, we recommend that the GLIM criteria be used as minimum nutritional assessment items for adults, also including a detailed comprehensive nutritional assessment in the presence of malnutrition. The use of this additional assessment may be beneficial for developing effective nutritional interventions. Future studies should provide information on how the nutritional status of patients with dysphagia is influenced by the implementation of a qualified nutritional assessment worldwide.

## Figures and Tables

**Table 1:** Nutritional assessment items used for adult patients with dysphagia.

Broad Category	Subcategory	Number of Articles	Disease, (*n*)	First Author	Year
Body mass index	Body mass index	9 [35,36,41,42,43,44,45,46,47]	Stroke (5), HNC (2), Amyotrophic lateral sclerosis (2), Pulmonary disease (2), Cardiovascular disease (2), Firearm injury (2), Cervical trauma (2), Diabetes mellitus (2), Dyslipidemia (2), Hypertension (2), Machado–Joseph disease (2), Meyge’s syndrome (2), Rubinstein–Taybi syndrome (2), Parkinson’s disease (2), Alzheimer’s disease (2), Dementia, Esophageal cancer, Brain tumor, Myelitis, Huntington’s disease, Progressive supranuclear palsy, Trigeminal neuropathy, Traumatic brain injury, Presbyphagia	da Silva AF, Barni GC	2020
				Maeda K, Ikenaga Y	2017
				Toh Yoon EW, Nakadate A	2016
				Ortega O	2015
				Lecleire S	2006
				Jacobsson C	1997
Nutritional screening tool	MNA-SF	4 [39,42,45,48]	Stroke	Nakazawa Y	2020
				Vilardell N, Maeda K	2017
				Ortega O	2015
Anthropometric measurements	Weight	5 [37,38,46,49,50]	Esophageal cancer (2), Stroke, HNC, Lung cancer	Kim J	2018
				Smith ZL	2017
				Wang YJ	2014
				Lecleire S	2006
				Elmståhl S	1999
	TSFT	1 [47]	Stroke, brain tumor	Jacobsson C	1997
	MAMC	1 [47]	Stroke, brain tumor	Jacobsson C	1997
Body composition	SMM (BIA)	1 [48]		Nakazawa Y	2020
	Percent body fat	1 [50]	Stroke	Elmståhl S	1999
	Lean body mass	1 [50]	Stroke	Elmståhl S	1999
Dietary assessment	Food intake level	2 [51,52]	Stroke, Brain trauma, Encephalitis, Central pontine myelinolysis, Neoplasm	Bülow M	2008
				Bartolome G	1997
	Food Frequency Questionnaire	1 [41]	Parkinson’s disease, Alzheimer’s disease, Huntington’s disease, Amyotrophic lateral sclerosis, Machado–Joseph disease, Meyge’s syndrome, Progressive supranuclear palsy, Stroke, Trigeminal neuropathy, Myelitis, Rubinstein–Taybi syndrome, Firearm injury, Cervical trauma, Presbyphagia, Diabetes mellitus, Hypertension, Dyslipidemia, Cardiovascular disease	Barni GC	2020
	Energy intake	1 [42]		Maeda K	2017
	Period to meal resumption and dietary form	1 [53]		Kishimoto N	2016
	Daily food intake	1 [40]	Stroke	Masiero, S	2008
Others	MNA	1 [54]	Alzheimer’s Disease	Tang Y	2017
	O-PNI	1 [43]		Toh Yoon EW	2016

Abbreviations: HNC, head and neck cancer; MNA-SF, Mini Nutritional Assessment-Short Form; MNA, Mini Nutritional Assessment; TSFT, Triceps skinfolds thickness; MAMC, mid-arm muscle circumference (MAMC = mid-upper arm circumference − π × TSFT); SMM, skeletal muscle mass; BIA, bioelectric impedance analysis; O-PNI, Onodera’s Prognostic Nutritional Index.

**Table 2:** Nutritional assessment items related to blood biomarkers for adult patients with dysphagia.

Broad Category	Subcategory	Number of Articles	Disease, (*n*)	First Author	Year
Blood biomarkers	Albumin	9 [43,44,46,47,49,50,54,55,56]	Stroke (3), Esophageal cancer (2), Oropharyngeal cancer, Alzheimer’s disease, Gaucher disease, Niemann–Pick disease, High cervical spinal cord injury, Brain tumor	Kimura Y	2019
				Smith ZL, Tang, Y	2017
				Toh Yoon EW, Nakadate A	2016
				Miyake N	2013
				Lecleire S	2006
				Elmståhl S	1999
				Jacobsson C	1997
	Hemoglobin	1 [54]	Alzheimer’s disease	Tang Y	2017
	Total protein	1 [55]	Gaucher disease, Niemann–Pick disease, High cervical spinal cord injury, Oropharyngeal cancer	Miyake N	2013
	Transferrin	1 [47]	Stroke, Brain tumor	Jacobsson C	1997
	Lymphocytes	1 [56]		Kimura Y	2019
	Pre-albumin	1 [47]	Stroke, Brain tumor	Jacobsson C	1997
	C-reactive protein	1 [50]	Stroke	Elmståhl S	1999
	Ceruloplasmin	1 [50]	Stroke	Elmståhl S	1999
	Transthyretin	1 [50]	Stroke	Elmståhl S	1999
	Retinol-binding protein	1 [50]	Stroke	Elmståhl S	1999
	Total iron-binding capacity	1 [50]	Stroke	Elmståhl S	1999
	Orosomucoid	1 [50]	Stroke	Elmståhl S	1999

**Table 3:** Nutritional assessment items used for adult patients with dysphagia in acute and post-acute settings.

	Acute Setting, (*n*)	Post-Acute Setting, (*n*)
Disease	Parkinson’s Disease, Alzheimer’s Disease, Huntington’s Disease, Amyotrophic Lateral Sclerosis, Stroke, Machado–Joseph Disease, Meige Syndrome, Rubinstein–Taybi Syndrome, Progressive supranuclear palsy, Trigeminal neuropathy, Traumatic brain injury, Firearm Injury, Myelitis, Cervical Trauma, Systemic arterial hypertension, Pneumonia, Diabetes mellitus, Dyslipidemia, Cardiovascular disease, Chronicobstructive pulmonary disease, Presbyphagia, Gaucher disease, Niemann-Pick disease, High cervical spinal cord injury, Oropharyngeal cancer, Head and Neck cancer, Esophageal cancer, Brain tumor, Lung cancer, Nasopharyngeal carcinoma	Stroke, Brain trauma, Encephalitis, Central pontine myelinolysis, Neoplasm, Alzheimer’s Disease
Body mass index	Body mass index (6)	Body mass index (3)
Nutritional screening tool	MNA-SF (2)	MNA-SF (2)
Anthropometric measurements	Weight (5), TSFT (1), MAMC (1)	none
Body composition	Percentage body fat (1), Lean body mass (1)	SMM (BIA) (1)
Dietary assessment	Food Frequency Questionnaire (1), Energy intake (1), Period to meal resumption and dietary form (1), Daily food intake (1)	Food intake level (2)
Blood biomarkers	Albumin (6), Total protein (1), Transferrin (1), Lymphocytes (1), Pre-albumin (1), C-reactive protein (1), Ceruloplasmin (1), Transthyretin (1), Retinol-binding protein (1), TIBC (1), Orosomucoid (1)	Albumin (3), Hemoglobin (1)
Others	none	MNA (1), O-PNI (1)

Abbreviations: MNA-SF, Mini Nutritional Assessment-Short Form; TSFT, Triceps skinfolds thickness; MAMC, mid-arm muscle circumference (MAMC = mid-upper arm circumference − π × TSFT); SMM, skeletal muscle mass; BIA, bioelectric impedance analysis; TIBC, total iron binding capacity; MNA, Mini Nutritional Assessment; O-PNI, Onodera’s Prognostic Nutritional Index.

## Supplementary Materials

The following are available online at https://www.mdpi.com/2072-6643/13/3/778/s1, Table S1: Nutritional assessment used for adult patients with dysphagia, Table S2: Sources excluded following full-text review.
