# Supplementary material for: Nutritional Assessment in Adult Patients with Dysphagia: A Scoping Review
Source: Nutrients. 2021 Feb 27;13(3):778. doi: 10.3390/nu13030778 (PMC7997289; doi:10.3390/nu13030778)
Supplement: Supplementary file 1 [file nutrients-13-00778-s001.pdf]

Supplementary Materials Table S1. Nutritional assessment used for adult patients with dysphagia.

Broad category	Subcategory	number of articles	Assessor	Age, years (range)*	Disease	Author, Year, Country
BMI	BMI	9	none	61.3 (IQR: 58.4–64.2)	Parkinson's Disease, Alzheimer's Disease, Amyotrophic Lateral Sclerosis, Stroke, Machado-Joseph Disease, Meige Syndrome Rubinstein–Taybi Syndrome, Traumatic brain injury, Injury by Firearm, Cervical Trauma, head and neck cancer, systemic arterial hypertension, pneumonia, diabetes mellitus, dyslipidemia, cardiovascular disease and chronicobstructive pulmonary disease	da Silva AF, 2020, Brazil [35]
			trained professionals	60.1±15.3	Parkinson's, Alzheimer's, Huntington's, Amyotrophic lateral sclerosis, Machado–Joseph, progressive supranuclear palsy, Meyge's syndrome, stroke, trigeminal neuropathy, Rubinstein-Taybi syndrome, myelitis, firearm injury, cervical trauma, presbyphagia, diabetes mellitus, hypertension, dyslipidemia and cardiovascular disease	Barni GC, 2020, Brazil [41]
			multidisciplinary team	COI: 72.9±11.4, ICOI: 78.9±8.3	stroke	Ikenaga Y, 2017, Japan [36]
			none	84.3±7.5	none	Maeda K,2017, Japan [42]
			none	76.6±10.5	none	Toh Yoon EW, 2016, Japan [43]
			none	72.1±11.0	Subacute Stroke	Nakadate A, 2016, Japan [44]
			multidisciplinary	Group1: 82, Group2: 80,	none	Ortega O, 2015, Spain [45]

			team	Group3: 81, Group4: 76		
			none	68	esophageal cancer	Lecleire S,2006, France [46]
			none	range 42–79	acute stroke, brain tumor	Jacobsson C, 1997, Sweden [47]
Nutritional screening tool	MNA-SF	4	none	men, 79.12±6.73<br>women, 79.88±7.82	none	Nakazawa Y, 2020, Japan [48]
			none	76.7±9.3	stroke	Vilardell N, 2017, Spain [39]
			none	84.3±7.5	none	Maeda K, 2017, Japan [42]
			multidisciplinary team	Group1: 82, Group2: 80, Group3: 81, Group4: 76	none	Ortega O, 2015, Spain [45]
Anthropometric measurements	Weight	5	none	SEMS group:62.4,<br>PEG group: 58.5	lung cancer	Kim J, 2018, Korea [37]
			none	59.1	esophageal cancer	Smith ZL, 2017, USA [49]
			none	54	nasopharyngeal carcinoma	Wang YJ, 2014, China [38]
			none	68	esophageal cancer	Lecleire S, 2006, France [46]
			none	74.8±8.4 (53–89)	stroke	Elmståhl S, 1999, Sweden [50]
	TSF	1	none	range 42–79	acute stroke, brain tumor	Jacobsson C, 1997, Sweden [47]
	MAMC	1	none	range 42–79	acute stroke, brain tumor	Jacobsson C, 1997, Sweden [47]
Body composition	SMM (BIA)	1	none	men, 79.12±6.73<br>women, 79.88±7.82	none	Nakazawa Y, 2020, Japan [48]
	Percentage body fat	1	none	74.8±8.4 (53–89)	stroke	Elmståhl S, 1999, Sweden [50]
	Lean body mass	1	none	74.8±8.4 (53–89)	stroke	Elmståhl S, 1999, Sweden [50]

Dietary assessment	Food intake level	2	none	Intervention: 70, control: 71	stroke	Bülow M, 2008, Sweden, Netherlands and France [51]
			none	61 (26–81)	Stroke, neoplasm, brain trauma, Encephalitis, central pontine myelinolysis	Bartolome G, 1997, Germany [52]
	Food Frequency Questionnaire	1	none	60.1±15.3	Parkinson’s, Alzheimer’s, Huntington’s, Amyotrophic lateral sclerosis, Machado-Joseph, progressive supranuclear palsy, Meyge’s syndrome, stroke, trigeminal neuropathy, Rubinstein–Taybi syndrome, myelitis, firearm injury, cervical trauma, presbyphagia, diabetes mellitus, hypertension, dyslipidemia and cardiovascular disease	Barni GC, 2020, Brazil [41]
	Energy intake	1	none	84.3±7.5	none	Maeda K,2017, Japan [42]
	Period to meal resumption and dietary form	1	none	72.7±8.6	none	Kishimoto N, 2016, Japan [53]
	Daily food intake	1	none	72.9	stroke	Masiero, S, et al, 2008, Italy [40]
Blood biomarkers	Albumin	9	none	84 (63–84)	none	Kimura Y, 2019, Japan [56]
			none	59.1	esophageal cancer	Smith ZL, 2017, USA [49]
			none	intervention: 72.5±2.5, control: 76.2±2.3	Alzheimer’s Disease	Tang Y, 2017, China [54]
			none	76.6±10.5	none	Toh Yoon EW, 2016, Japan [43]
			none	72.1±11.0	Subacute Stroke	Nakadate A, 2016, Japan [44]

			none	65.6 (15–91)	Gaucher disease, Niemann-Pick disease, High cervical spinal cord injury, Oropharyngeal cancer	Miyake N, 2013, Japan [55]
			none	68	esophageal cancer	Lecleire S, 2006, France [46]
			none	74.8±8.4 (53–89)	stroke	Elmståhl S, 1999, Sweden [50]
			none	range 42–79	acute stroke, brain tumor	Jacobsson C, 1997, Sweden [47]
	Hemoglobin	1	none	intervention: 72.5±2.5, control: 76.2±2.3	Alzheimer's Disease	Tang Y, 2017, China [54]
	Total protein	1	none	65.6 (15–91)	Gaucher disease, Niemann–Pick disease, High cervical spinal cord injury, Oropharyngeal cancer	Miyake N, 2013, Japan [55]
	Transferrin	1	none	range 42–79	acute stroke, brain tumor	Jacobsson C, 1997, Sweden [47]
	Lymphocytes	1	none	84 (63–84)	none	Kimura Y, 2019, Japan [56]
	Pre-albumin	1	none	range 42–79	acute stroke, brain tumor	Jacobsson C, 1997, Sweden [47]
	C-reactive protein	1	none	74.8±8.4 (53–89)	stroke	Elmståhl S, 1999, Sweden [50]
	Ceruloplasmin	1				
	Transthyretin	1				
	Retinol-binding protein	1				
	TIBC	1				
	Orosomucoid	1				
Others	MNA	1	A trained dietitian	intervention: 72.5±2.5, control: 76.2±2.3	Alzheimer's Disease	Tang Y, 2017, China [54]

O-PNI	1	none	76.6±10.5	none	Toh Yoon EW, 2016, Japan [43]

Abbreviations: BMI, body mass index; IQR, interquartile range; COI, complete oral intake; ICOI, incomplete oral intake; MNA-SF, Mini Nutritional Assessment-Short Form; TSF, triceps skinfolds thickness; USA, United State of America; PEG, percutaneous endoscopic gastrostomy; MAMC, mid-arm muscle circumference (MAMC=MUAC- π×TSF); SMM, skeletal muscle mass; BIA, bioelectric impedance analysis; SEMS, self-expandable metallic stent; TIBC, total iron binding capacity; MNA, Mini Nutritional Assessment; O-PNI, Onodera's Prognostic Nutritional Index. *: Age is the average of the participants' age without any comments.

Supplementary Materials Table S2. Sources excluded following full-text review.

Reasons	Title	Author, Year, Country
The diagnosis of dysphagia is made clinically using methods other than videofluoroscopy and fiberoptic endoscopy.	Long-Term Nasogastric Versus Percutaneous Endoscopic Gastrostomy Tube Feeding in Older Asians With Dysphagia: A Pragmatic Study	Jaafar MH, 2019, Malaysia [73]
	Comparison of enteral nutrition with total parenteral nutrition for patients with locally advanced unresectable esophageal cancer harboring dysphagia in definitive chemoradiotherapy	Furuta M,.2019, Japan [74]
	The Prevalence and Prognosis of Sarcopenic Dysphagia in Patients Who Require Dysphagia Rehabilitation	Wakabayashi H, 2019, Japan [75]
	Effect of an oral health programme on oral health, oral intake, and nutrition in patients with stroke and dysphagia in Taiwan: A randomised controlled trial	Chen HJ, 2019, Taiwan [76]
	Design and implementation of modified-texture diet in older adults with oropharyngeal dysphagia: a randomized controlled trial	Reyes-Torres CA, 2019, Mexico [77]
	Comparison of long-term outcomes between enteral nutrition via gastrostomy and total parenteral nutrition in older persons with dysphagia: A propensity-matched cohort study	Masaki S, 2019, Japan [78]
	The effects of resistance training of swallowing muscles on dysphagia in older people: a cluster, randomized, controlled trial	Wakabayashi H, 2018, Japan [79]

	Effect of a Minimal-Massive Intervention in Hospitalized Older Patients with Oropharyngeal Dysphagia: A Proof-of-Concept Study	Martín, A, et al. 2018, Spain [80]
	Cognitive impairment has no impact on hospital-associated dysphagia in aspiration pneumonia patients	Maeda K, 2018, Japan [81]
	Malnutrition risk predicts recovery of full oral intake among older adult stroke patients undergoing enteral nutrition: Secondary analysis of a multicentre survey (the APPLE study)	Nishioka S, 2017, Japan [82]
	Nutritional status of older patients with oropharyngeal dysphagia in a chronic versus an acute clinical situation	Carrión S,2017, Spain [57]
	LOW SERUM CHROMIUM IS RARE IN PATIENTS THAT UNDERWENT ENDOSCOPIC GASTROSTOMY FOR LONG TERM ENTERAL FEEDING	Santos CA, 2017, Portugal [83]
	A Dedicated Nutritional Care Program (NUTRICARE) to reduce malnutrition in institutionalised dysphagic older people: A quasi-experimental study.	Zanini M, 2017, Italy [84]
	Factors associated with the level of oral intake in hospitalized older adults with dysphagia: The importance of mental activity	Kuroda Y, 2016, Japan [85]
	Normalcy of food intake in patients with head and neck cancer supported by combined dietary counseling and swallowing therapy: A randomized clinical trial	Van Den Berg MGA, 2016, Netherlands [86]
	Malnutrition and Clinical Outcome of 234 Head and Neck Cancer Patients who Underwent Percutaneous Endoscopic Gastrostomy	Fonseca J, 2016, Portugal [87]
	Effects of a feeding intervention in patients with Alzheimer's disease and dysphagia	Chen LL,2016, China [88]
	Clinical Variables Associated with Hydration Status in Acute Ischemic Stroke Patients with Dysphagia	Crary MA, 2016, USA [89]
	Serum trace elements in dysphagic gastrostomy candidates before	Santos CA, 2016, Portugal [90]

	endoscopicgastrostomy for long term enteral feeding	
	Swallowing rehabilitation with nutrition therapy improves clinical outcome in patients with dysphagia at an acute care hospital	Iwamoto M, 2014, Japan [91]
	Nutritional factors associated with survival following enteral tube feeding in patients with motor neuron disease	Rio A, 2010, United Kingdom [92]
	Serum trace elements in tube-fed neurological dysphagia patients correlate with nutritional indices but do not correlate with trace element intakes: Case of patients receiving enough trace elements intake	Obara H, 2008, Japan [93]
	The effectiveness of targeted feeding assistance to improve the nutritional intake of elderly dysphagic patients in hospital	Wright L, 2008, United Kingdom [94]
	A prospective comparison of percutaneous endoscopic gastrostomy and nasogastric tube feeding in patients with acute dysphagic stroke	Hamidon, BB, 2006, Malaysia [95]
	Nutritional Status and Quality of Life in Patients with Percutaneous Endoscopic Gastrostomy (PEG) in Practice: Prospective One-Year Follow-Up	Klose J, 2003, Germany [96]
	Eating difficulties, need for assisted eating, nutritional status and pressure ulcers in patients admitted for stroke rehabilitation	Westergren A, 2001, Sweden [97]
	Dysphagia and nutritional status in multiple sclerosis	
	A randomised prospective comparison of percutaneous endoscopic gastrostomy and nasogastric tube feeding after acute dysphagic stroke	Thomas FJ, 1999, United Kingdom [98]
	Complications and outcome after acute stroke: Does dysphagia matter?	Norton B, 1996, United Kingdom [99]
Not all participants are dysphagic patients.	Low level of phosphate in male patients reporting swallowing disturbances in early Parkinson's disease	Smithard DG, 1996, United Kingdom [100]
	Relationship Between Body Mass Index and Rehabilitation Outcomes in Subacute	Håglin L, 2020, Sweden [101]

	Stroke with Dysphagia.	
	Dysphagia is associated with oral, physical, cognitive and psychological frailty in Japanese community-dwelling elderly persons.	Morone G, 2020, Italy [102]
	Dysphagia is more strongly associated with increased intramuscular adipose tissue of the quadriceps than with loss of muscle mass in older inpatients	Nishida N, 2020, Japan [103]
	A Significant Association of Malnutrition with Dysphagia in Acute Patients	Akazawa N, 2019, Japan [104]
	Survival in older adults with dementia and eating problems: to PEG or not to PEG?	Saito T, 2018, Japan [105]
	Neck circumference is not associated with dysphagia but with undernutrition in elderly individuals requiring long-term care	Ticinesi A, 2016, Italy [106]
	Head lifting strength is associated with dysphagia and malnutrition in frail older adults	Wakabayashi H, 2016, Japan [107]
	Oral cancer malnutrition impacts weight and quality of life	Wakabayashi H, 2015, Japan [108]
	Effect of induction chemotherapy on swallow physiology and saliva production in patients with head and neck cancer: A pilot study	Gellrich NC, 2015, Germany, Austria, Switzerland [109]
	Impact of early enteral nutrition on short term prognosis after acute stroke	Mittal BB, 2015, USA [110]
	Eating difficulties among patients 3months after stroke in relation to the acute phase	Zheng T, 2015, China [111]
	Prospective evaluation of oro-pharyngeal dysphagia after severe traumatic brain injury.	Medin J, 2012, Sweden [112]
	Evaluation of nutritional status in children with refractory epilepsy	Terré R, 2007, Spain [113]
	Pre-and postoperative nutritional evaluation in patients with chagasic megaesophagus	Bertoli S, Italy [114]
	Gastrooesophageal reflux in children with cerebral palsy.	Penhavel FA, 2004, Brazil [115]
No nutritional assessment	Safety and efficacy of functional laryngectomy for end-stage dysphagia	Reyes A. L, 1993, United Kingdom [116]
	Effects of device-facilitated isometric progressive resistance oropharyngeal therapy on swallowing and health-related outcomes in older adults with dysphagia	Topf M. C, 2018, USA [117]
	Dysphagia assessed by the 10-item Eating Assessment Tool is associated with nutritional	Rogus-Pulia N, 2016, USA [118]

	status and activities of daily living in elderly individuals requiring long-term care	
	Efficacy of concurrent chemoradiotherapy as a palliative treatment in stage IVB esophageal cancer patients with dysphagia	Wakabayashi H, 2016, Japan [17]
	Using different perspectives to generate items for a new scale measuring medical outcomes of dysphagia (MOD)	Ikeda E, 2011, Japan [119]
Participants are not adults	Parent-reported indicators for detecting feeding and swallowing difficulties and undernutrition in preschool-aged children with cerebral palsy.	Martino R, 2009, Canada [120]
	Diagnosis and treatment of feeding disorders in children with developmental disabilities	Benfer KA, 2017 [121]
	Oropharyngeal dysfunction and gastroesophageal dysmotility are present in girls and women with Rett syndrome	Schwarz SM, 2001 [122]
Non-English language	Factors associated with mortality in patients with dysphagia help in making dietary and nutritional choices	Motil KJ, 1999 [123]
	Percutaneous endoscopic gastrostomy in patients with ENT tumors	Ferrero L, 2015, Spain [124]
Review articles	Nutritional management in home care: Including eating disorder and dysphagia assessments	Motsch C, 1998, Germany [125]
